# Cell communication and protein degradation: All in one parasitic package

**DOI:** 10.1002/jev2.12116

**Published:** 2021-07-05

**Authors:** Michal Sharon, Neta Regev‐Rudzki

**Affiliations:** ^1^ Department of Bimolecular Sciences Weizmann Institute of Science Rehovot Israel

A *parasite* is a unicellular or multicellular organism that is dependent on another organism (host) for its survival and proliferation. The parasite benefits from a prolonged association with its host (Loker & Hofkin, 2015), as without it, the parasite cannot grow and multiply. Thus, parasites must keep their host alive for as long as possible without killing it, yet their infection may cause diseases and, in some cases, like malaria, even mortality.

Three main classes of parasites can cause disease in humans: protozoa, helminths and ectoparasites. The former are unicellular eukaryotic parasites (e.g., *Plasmodium falciparum* and *Trypanosoma brucei*), whereas the helminths are parasitic worms (e.g., *Schistosoma haematobium* and *Fasciola hepatica*), and the ectoparasites, organisms that infest the host skin (e.g., *Haemaphysalis longicornis* and *Sarcoptes scabiei*). These classes of parasites are master manipulators whose life cycles feature unique adaptions, involving sophisticated strategies to alter the host environment, including the secretion of multipurpose extracellular vesicles (EVs) (Coakley et al., 2015; Ofir‐Birin & Regev‐Rudzki, 2019).

EVs are heterogeneous in terms of size (30–500 nm in diameter) and transfer functional signals to target cells by carrying a cornucopia of different molecules, such as proteins, glycans, lipids, RNA and DNA (Schorey et al., 2015; Tkach & Théry, 2016). Since the release of EVs is an integral part of a parasite's life cycle and course of the infection, it stands to reason that these organelles are essential for their survival. Indeed, these shuttling vesicles provide a robust delivery system to facilitate parasitic growth and development, the transfer of virulence factors, adherence to host tissues, and evasion of immune responses (Mardahl et al., 2019; Ofir‐Birin et al., 2017). They effectively manipulate the host's immune system by inhibiting or activating responses as well as by affecting a variety of other (non‐immune) target human cells (Marcilla et al., 2014; PMID: 29577413 by Hosseini‐Beheshti, E : Ofir‐Birin & Regev‐Rudzki, 2019).

A case in point is the malaria parasite *Plasmodium (P) falciparum*, which secretes EVs while residing in human red blood cells (RBCs) that affect a range of target host cells (Mantel et al., 2016; Mantel et al., 2013; Regev‐Rudzki et al., 2013; Sisquella et al., 2017; Ye et al., 2018). Intriguingly, our recent investigation of the protein content of these secreted EVs revealed great enrichment in parasitic and host subunits of the proteasome degradation complex (Dekel et al., 2021). Our results indicate that these proteasome subunits are assembled within the EVs into intact, functional 20S proteasomes, and that the activities of these encapsulated complexes promote parasitic growth. In particular, we found that following *P. falciparum‐*derived EV introduction, two sequential steps take place. First, RBC host proteins undergo specific phosphorylation events, including in several cytoskeletal proteins. Second, the delivered 20S proteasomes mediate the degradation of the phosphorylated cytoskeleton proteins by a ubiquitin‐independent process (Dekel et al., 2021). The result is a reduction in the stiffness of the naïve RBCs’ membrane, thus, priming them for parasitic invasion, with direct implications for the parasite's growth capacity.

The proteasome is a conserved degradation machinery that is vital for maintaining proteostasis by irreversibly removing misfolded, damaged or short‐lived regulatory proteins (Baumeister et al., 1998). Two alternative proteasomal degradation mechanisms, which are not mutually exclusive, exists in cells, involving the 26S and 20S proteasome complexes (Goldberg, 2003; Kumar Deshmukh et al., 2019). The 26S proteasome comprises a 19S regulatory particle, which recognises ubiquitin‐tagged substrates, and a 20S catalytic core particle, where substrates are degraded via the breakage of peptide bonds. Degradation by the 26S proteasome, which is the major cellular degradation route, is an ATP‐dependent process that is coordinated by three different types of enzymes (E1, E2, and E3) that ubiquitinate the substrate and sensitize it to degradation (Hershko & Ciechanover, 1998). In contrast, the 20S proteasome, on its own, can degrade protein substrates in a ubiquitin‐ and ATP‐independent manner, by cleaving unfolded or unstructured regions within its substrates (Ben‐Nissan & Sharon, 2014; Kumar Deshmukh et al., 2019).

Hence, the preference of *P. falciparum*‐derived EVs for 20S‐proteasome‐mediated degradation over the 26S proteasome pathway may arise from the simplicity and self‐reliance of the 20S system. Moreover, the size of the malaria‐derived EVs, which mostly ranges between 50 and 200 nm in diameter, likely restricts the encapsulation of 26S proteasomes (∼45 × 20 nm) to a greater degree than that of 20S particles (∼15 × 12 nm).

**FIGURE 1:**
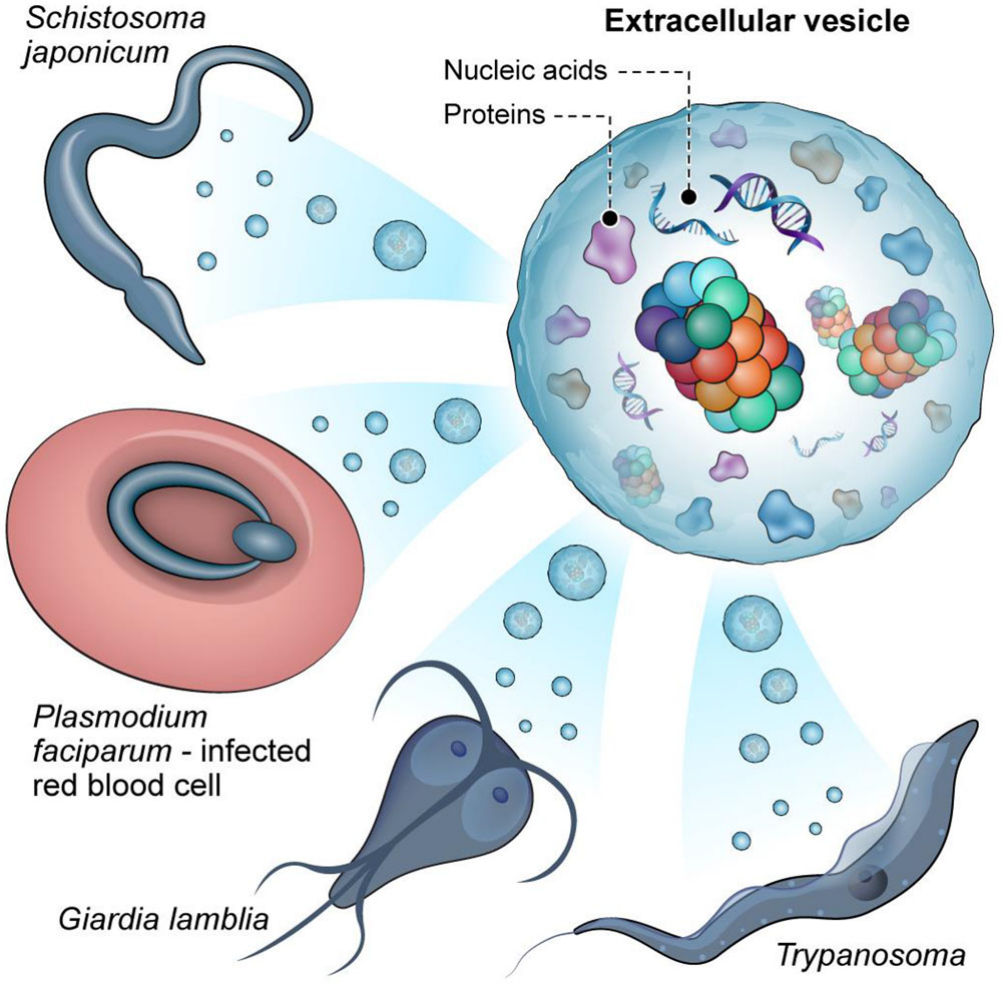
20S proteasome subunits were identified in EVs secreted from eukaryotic parasites. Many aspects regarding the role played by these EV proteasome complexes remain enigmatic

With the rising interest in parasite‐derived EV content, worldwide proteomic initiatives have started to expose the composition of their protein cargo. An interesting observation that arises from the accumulated data is that the three main classes of parasites secrete 20S proteasome subunits (Figure 1). With respect to the helminths, 20S proteasome subunits were found encapsulated in the EVs of *Trichuris muris* (Eichenberger et al., 2018)*, Fasciola hepatica* (Cwiklinski et al., 2015)*, Schistosoma japonicum* (Du et al., 2020; Zhu et al., 2016), *Schistosoma haematobium* (Mekonnen et al., 2020) and  *Echinococcus granulosus* (Nicolao et al., 2019). 20S proteasome subunits were also identified in EVs secreted by the protozoa *Giardia lamblia* (Stefanic et al., 2006), *Trichomonas vaginalis* (Nievas et al., 2018), *Leishmania donovani* (Silverman et al., 2010), *Leishmania major* (Atayde et al., 2015)*, Leishmania infantum* (Douanne et al., 2020), *Toxoplasma gondii* (Wowk et al., 2017) and *Acanthamoeba castellanii* (Lin et al., 2019). Similar results were found in *Haemaphysalis longicornis* (Nawaz et al., 2020), representing the third parasite class, ectoparasites. Even in EVs from non‐parasitic pathogens, such as the fungus *Candida albicans* (Martinez‐Lopez et al., 2020), *Histoplasma capsulatum* (Albuquerque et al., 2008) and the mycobacteria *Mycobacterium tuberculosis* (Lee et al., 2015), the presence of 20S proteasome subunits was identified. Moreover, 26S proteasome subunits were also identified in a minority of the above‐noted studies (seven out of 20). Given the broad range of parasites whose EVs contain proteasomes, our results hint at a more general biological principle, in which parasites deliver the more ‘compact’ degradation system, namely the 20S proteasome, to facilitate their invasion, growth and development.

It is important to note that the presence of proteasome subunits within the various pathogen‐secreted vesicles does not necessarily indicate that an assembled and active complex is being delivered. In the case of the *P. falciparum*‐derived EVs, this aspect was specifically investigated, leading to the understanding that the EVs indeed harbor an intact and active 20S proteasome complex comprising the three enzymatic activities, that is, caspase‐like, chymotrypsin‐like and trypsin‐like. Whether this is a general phenomenon is still not known, as the assembly state of the proteasome in other parasite‐derived EVs remains to be determined.

Further support for the contributing role of proteasomes in parasitemia comes from accumulating evidence indicating that proteasome inhibitors affect parasitic growth. For example, in the case of *Acanthamoeba castellanii*, the well‐recognized agent of a fatal brain disease, the proteasome inhibitor bortezomib was shown to abolish extracellular proteolytic activities, affect parasite growth and block parasite encystation and excystation (Siddiqui et al., 2016). The growth of *Entamoeba histolytica* is also affected in a dose‐dependent manner by proteasome inhibitors (Makioka et al., 2002). Similarly, proteasome inhibition irreversibly inhibits *Entamoeba invadens* encystation, suggesting that the ability of the trophozoites to encyst is lost during exposure to the drug (González et al., 1997; Makioka et al., 2002). Helminths were also shown to be sensitive to proteasome inhibitor treatment (Guerra‐Sá et al., 2005). For instance, proteasome inhibition affects the blood fluke *Schistosoma mansoni*, reducing the number of lung stage schistosomula and worm burden and, consequently, decreasing the egg output in infected mice (Guerra‐Sá et al., 2005). The effect of proteasome inhibitors on the growth and replication of protozoan parasites has been described for *Leishmania mexicana* (Robertson, 1999), *Trypanossoma cruzi* (De Diego et al., 2001), *Trypanossoma brucei* (Mutomba et al., 1997, Nkemgu‐Njinkeng et al., 2002)*, Plasmodium berghei* (Gantt et al., 1998)*, Plasmodium falciparum* (Kirkman et al., 2018) and *Toxoplasma gondii* (Shaw et al., 2000). Fungi showed a comparable response: In *Candida albicans*, proteasome inhibition induces filamentation (Hossain et al., 2020), while in n *Cryptococcus neoformans*, it leads to a decrease in both capsule production and fungal growth (Geddes et al., 2016). Taken together, these studies hint toward a general impact of proteasome inhibitors on parasitic growth. However, there still remains a gap in knowledge as to whether these described parasitic effects are due to the inhibition of the EV‐20S proteasome, as witnessed for *Plasmodium falciparum* (Dekel et al., 2021).

The key question that arises is, why do parasites transport 20S proteasomes within their secreted vesicles. Since parasite‐derived EVs mediate environmental adaptation within their host (Ofir‐Birin & Regev‐Rudzki, 2019), it is likely that the proteasome complex assists in this process. One possibility is that the secreted proteasomes help initiate the first stage of infection. This was found to be the case in *P. falciparum*. This parasite primes naïve RBCs for parasite invasion by transferring, via EVs, 20S proteasome complexes to healthy RBCs that degrade cytoskeleton proteins and, thereby, reduce the stiffness of the RBC membrane (Dekel et al., 2021). It could also be that the proteolytic activity of the released proteasome promotes the adherence of the parasite to its target cells and aids colony establishment in these niches. Another possible impact is indicated by the mounting evidence that EVs promote cooperative behaviour in parasites. During infection, parasites do not act solely as ‘selfish’ individuals, but rather often use EVs to mediate behaviour as integrated communities, for instance, to coordinate their movement as a group (social motility) (Eliaz et al., 2017), promote their sexual development (Mantel et al., 2013; Regev‐Rudzki et al., 2013) or facilitate the transfer of a virulence factor between them (Szempruch et al., 2016). Proteasome delivery between parasites may also extend the functional capacity of parasitic proteins through 20S proteasome‐mediated proteolytic processing, as has been shown for human proteins (Olshina et al., 2018, Solomon et al., 2017). The proteasome may also be involved in the vesicle release process itself, as was shown for activated human T‐lymphocytes (Tucher et al., 2018). This key aspect of EV‐20S deserves further attention, and will likely provide more than a single answer.

Can we implement this knowledge of parasitic EV proteasome involvement in parasite infection as a novel intervention strategy to control parasites and the diseases they cause? The EV‐20S proteasomes present in body fluid biopsies could serve as a parasitic footprint to improve our ability to diagnose the infection in its different stages. Moreover, we expect that, in the future, efficient inhibition of the EV proteasome will indeed serve as a therapeutic avenue. Primary steps in this direction will be to determine whether the delivered proteasomes are active and whether they are composed solely of parasitic subunits or contain also host components. This knowledge is critical for generating selective EV‐20S proteasome compounds that sufficiently inhibit the parasitic complex rather than the human counterpart. Such a task is not trivial, given the high level of conservation of proteasome active sites in eukaryotes (Xie et al., 2019). However, progress in this direction has already been made with the design of compounds that show specificity against *P. falciparum* (Lin et al., 2023; Mata‐Cantero et al., 2019; Stokes et al., 2019) and *Trypanosoma brucei* proteasomes (Steverding et al., 2011), and we expect that additional selective compounds will arise.

Lastly, numerous clinical studies show that, 20S proteasome complexes circulate freely in blood plasma (Dwivedi et al., 2021). This observation raises the possibility that they may also be secreted by an additional yet unknown mechanism, which is not involving EVs, underscoring further need to investigate circulating 20S proteasomes in the context of parasitic infection.
